# R-CHOP intensification with mid-cycle methotrexate and consolidating AraC/TT with BCNU/aHSCT in primary aggressive lymphoma with CNS involvement

**DOI:** 10.1007/s00432-021-03663-x

**Published:** 2021-06-03

**Authors:** Maximilian J. Steinhardt, Franziska C. Krummenast, Andreas Rosenwald, Elena Gerhard-Hartmann, Anke Heidemeier, Hermann Einsele, Max S. Topp, Johannes Duell

**Affiliations:** 1 Medizinische Klinik und Poliklinik II, Universitätsklinikum Würzburg, Oberdürrbacher Straße 6, 97080 Würzburg, Germany; 2 Institut für Pathologie der Universität Würzburg, Universitätsklinikum Würzburg, Josef-Schneider-Straße 2, 97080 Würzburg, Germany; 3 Institut für Diagnostische und Interventionelle Radiologie, Universitätsklinikum Würzburg, Oberdürrbacher Straße 6, 97080 Würzburg, Germany; 4 Department of Internal Medicine II, University Hospital Würzburg, Oberdürrbacher Street 6, 97080 Würzburg, Germany

**Keywords:** Lymphoma, R-CHOP, MTX, HD

## Abstract

**Purpose:**

Patients suffering from aggressive systemic peripheral lymphoma with primary central nervous system involvement (PCL) are a rare and sparsely investigated population. Recommended treatment regimens include a combination of intrathecal and systemic chemotherapy as well as whole brain radiotherapy while offering relatively poor survival.

**Methods:**

We conducted a single-center retrospective study that analyzed safety and outcome of 4 + 4 cycles Rituximab (R)-CHOP and R-high-dose Methotrexate (HD-MTX) for newly diagnosed, transplant-eligible patients (“Ping-Pong”), followed by Cytarabine (AraC)/Thiotepa (TT), BCNU/TT, and autologous hematologic stem cell transplantation (aHSCT). We retrospectively analyzed a set of 16 patients with high–intermediate or high-risk IPI status.

**Results:**

Overall response rate to Ping-Pong was 100% measured by CT/MRI, including 93.75% complete remissions after BCNU/TT followed by PBSCT. One patient failed to qualify for high-dose chemotherapy due to progression when receiving Cytarabine/TT. All patients experienced grade III adverse events, 3 of them a grade IV adverse event. Estimated progression-free survival is 93.75% after a 4.8-year follow-up currently.

**Conclusion:**

Our study suggests high effectivity of R-CHOP with mid-cycle MTX with aHSCT consolidation towards acceptable OS results in this challenging patient population.

## Introduction

Diffuse large B-cell lymphoma (DLBCL) is the most common aggressive B-cell malignancy. Curative treatment is possible with R-CHOP and prognosis is evaluated on an age-adjusted IPI scoring system (aIPI). To this end, patients with an aIPI of zero have an excellent outcome of over 90% PFS at 5 years, whereas patients with an aIPI of 3–4 show a 5-year PFS of 40% (Cheson et al. 1999). For younger patients with an aIPI of 1–4, intensified protocols such as R-CHOEP, R-DA-EPOCH, or R-ACVB are applied resulting in a 5-year PFS of 50–60%. High-dose chemotherapy (HD) with autologous hematopoietic stem cell transplantation (aHSCT) has been offered to younger DLBCL patients, but has not established itself as standard (Pfreundschuh et al. 2006).

Primary central nervous system lymphoma (PCNSL) is recognized as separate identity, and is currently treated with blood–brain-barrier passing protocols such as high-dose MTX, TT, and AraC followed by HD with aHSCT in younger patients (Ferreri et al. 2016). This approach results in a PFS of 60% as reported by several study groups (Illerhaus et al. 2012; Omuro et al. 2015; Cote et al. 2012; Hollender et al. 2000; Schorb et al. 2013). A particular adverse situation occurs when DLBCL patients present with both nodal disease and CNS involvement. This finding can be seen in less than 4% of primary diagnosis of DLBCL (Akkas and Vural 2013), and no standardized approach how to treat such patients has been developed. The retrospective outcome for this group of patients remains inferior with a 3-year survival of 26% without, and 75% with HD + aHSCT (Damaj et al. 2015).

There is few prospective data about the specific subgroup. An Italian study reported forty patients, including 16 PCL patients. After a combination therapy including HD-MTX, HD cytarabine, and HD etoposide combined with rituximab and liposomal cytarabine, it found a 5-year OS rate of 41% (Ferreri et al. 2015a). The MARIETTA trial reported 79 patients, among those 32 PCL patients, both relapsed and therapy naive. After three courses of MATRix (AraC, TT and R) as well as three courses of R-ICE (R, ifosfamide, cisplatin, E), intrathecal therapy, and BCNU/TT + aHSCT, a 2-year OS of 42% was reported (Ferreri et al. 2021).

We present the whole cohort of 16 patients who presented at our center with SCL between 2013 and 2018. They were treated uniformly with a newly developed protocol, which includes an induction with 4 cycles of R-CHOP-21 and R-MTX on day 14 ("Ping-Pong"), followed by dose intensification with 2 cycles R-TT/AraC and BCNU/TT followed by aHSCT. Feasibility, toxicity and outcomes are included in this report, demonstrating a high rate of disease control with an OS of 93.75% at a median of 4.8 years with manageable toxicity profile.

## Methods

### Identification of patients

Sixteen patients (8 male, 8 female) with a median age of 60.2 [34.7–76.7] years at diagnosis that were treated at our center between March 2013 and June 2018 were retrospectively analyzed for this study. All patients were discussed prior to commencing treatment in our NHL tumor board, consisting of a pathologist, a radiotherapist, a specialist in nuclear medicine, radiologists, and hematologists. Treatment was offered only to HD-eligible patients with ECOG < 3. An ECOG rating of 3 due to lymphoma was accepted. An ethical review committee approved retrospective analysis and data acquisition.

A standard operating procedure at our center requires that every newly diagnosed DLBCL is staged via full body computed tomography (CT)-scan, bone marrow biopsy, and verification of diagnosis via central pathology review. DLBCL were classified as recommended by the WHO classification of 2016 (Swerdlow et al. 2016). All patients with clinical signs of CNS involvement or IPI > 2 received a cranial magnetic resonance tomography (MRI), and lumbar puncture with cerebrospinal fluid (CSF) and testing via cytology and flow cytometry. In addition, patients demonstrating potential dura involvement in CT scans were further evaluated via MRI of the involved region. Renal and hepatic assessment as well as HIV/Hepatitis diagnostics were performed in all subjects before chemotherapy. All patients had normal ejection fraction via echocardiography and normal lung function levels via spirometry.

Biopsy of the central nervous lymphoma manifestation was conducted in eight (50%) of all patients enrolled. Another seven patients were enrolled after confirming infiltration via MRI, even if CSF analysis did not show meningeosis. One patient, although negative in MRI, presented with central Horner Syndrome and was clinically suspected to have diffuse cerebral lymphoma infiltration. In patients with HIV-associated DLBCL, antiviral therapy was continued. Response assessment was performed according to the Lugano classification (Cheson et al. 2014).

We retrospectively analyzed response after induction, consolidation, and HD, for toxicity and time-dependent outcome such as duration of response (DOR), progression-free survival (PFS), and overall survival (OS).

### Treatment protocol and response assessment

Patients with high disease burden received a pre-phase of 100 mg Prednisone for 5 days after staging completion. Then, the induction protocol of four times standard R-CHOP-21 (Pfreundschuh et al. 2006) with R-MTX 4 g/m^2^ (Pfreundschuh et al. 2006) on day 14, followed by leucovorine rescue, was administered. Rituximab was given at 375 mg/m^2^ (Pfreundschuh et al. 2006) i.v. or 1400 mg s.c. In patients with proven CSF involvement, intrathecal therapy was performed with liposomal cytarabine weekly (Garcia-Marco et al. 2009) or triple therapy (MTX, AraC, and dexamethasone) three times a week additionally to systemic therapy. Intrathecal therapy was continued until no lymphoma cells were detected by both microscopic examination and flow cytometry.

Response assessment according to Lugano classification criteria (2008) was conducted 2–4 weeks after the last Ping-Pong cycle via CT scan and MRI of the involved region (Cheson et al. 2014). Another bone marrow puncture was conducted before stem cell harvest if there had been infiltration at diagnosis.

**Fig. 1:**
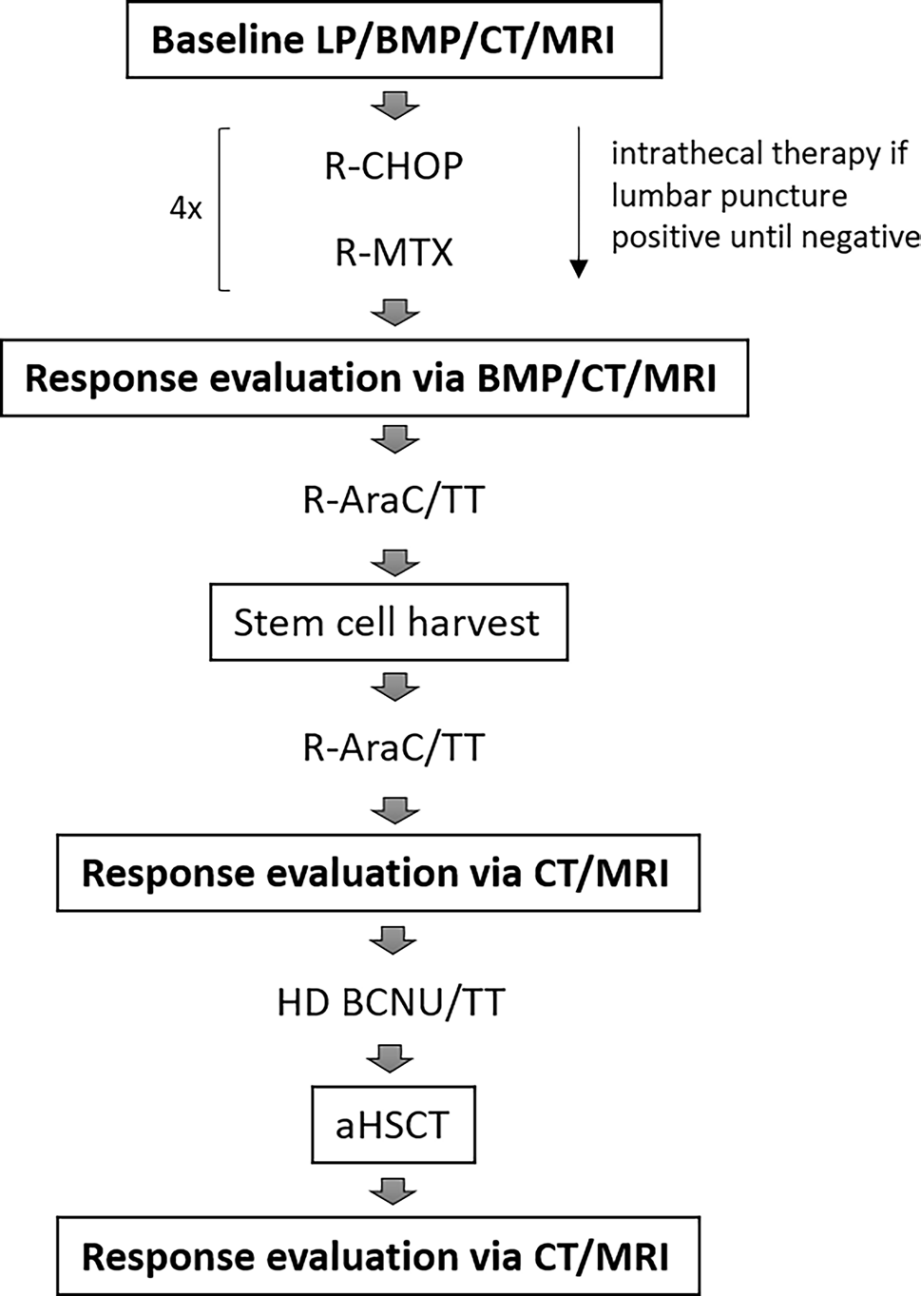
Treatment and staging. *LP* lumbar puncture, *BMP* bone marrow puncture, *CT* computed tomography, *cMRI* cranial magnetic resonance imaging, *R-CHOP* Rituximab, Cyclophosphamide, Vincristine, Prednisone, *MTX* methotrexate, *AraC* cytarabine, *TT* thiotepa, *HD* high dose, *BCNU* carmustine, *aHSCT* autologous hematopoietic stem cell transplantation

If at least a partial response or better was documented, the patient was eligible for dose intensification including HD with aHSCT. To this end, two cycles of R-AraC/TT, repeated after 21 days, were conducted. 10 µg/kg body weight G-CSF were given to support autologous stem cell collection after the first cycle. Stem cell collection was initiated when leucocyte count was > 2500/µl and CD34^+^ count > 20/nl. If those numbers could not be reached by G-CSF alone, we added Plerixafor at a daily dose of 0.24 mg/kg body weight. Harvest was considered sufficient when two transplants containing each > 2 × 10 (Cote et al. 2012) CD34^+^ cells/kg body weight were collected. One week after harvest, another cycle of R-AraC/TT was conducted. A second response assessment was performed 2–3 weeks after initiation of the second cycle of R-AraC/TT via CT scan, MRI, and bone marrow puncture as described. We initiated HD 1 week after leukocyte regeneration. One transplant contained > 2 × 10 (Cote et al. 2012) CD34^+^ cells/kg body weight. In Table 1, we give an overview of application mode and dosage; Fig. 1 shows the overall therapy regimen. A final response assessment was performed 4–6 weeks after transplantation via MRI/CT or PET-CT scan.

**Table 1:** Overview of chemotherapy regimen

R-CHOP		
Rituximab i.v./s.c	375 mg/m^2^/1400 mg abs	Day 1
Cyclophosphamide	750 mg/m^2^ i.v	Day 1
Doxorubicin	50 mg/m^2^ i.v	Day 1
Vincristin	2 / 1 mg abs. i.v	Day 1
Prednisone	100 mg abs. i.v	Days 1–5
R-MTX		
Rituximab i.v./s.c	375 mg/m^2^/1400 mg abs	Day 0
MTX	4 g/m^2^	Day 1
R-AraC/TT		
Rituximab s.c	1400 mg abs. s.c	Day 1
Cytarabine	4 g/m^2^	Days 1–^2
Thiothepa	40 mg/m^2^	Day ^2
R-HD-BCNU/TT		
Rituximab s.c	1400 mg abs	Day − 7
BCNU	400 mg/m^2^	Day − 6
Thiothepa	2 × 5 mg/m^2^	Day − 5/ − 4
aHSCT	> 2 × 10^6^ CD34^+^ cells/ kg body weight	Day 0

### Supportive care and standard procedures

**Fig. 2:**
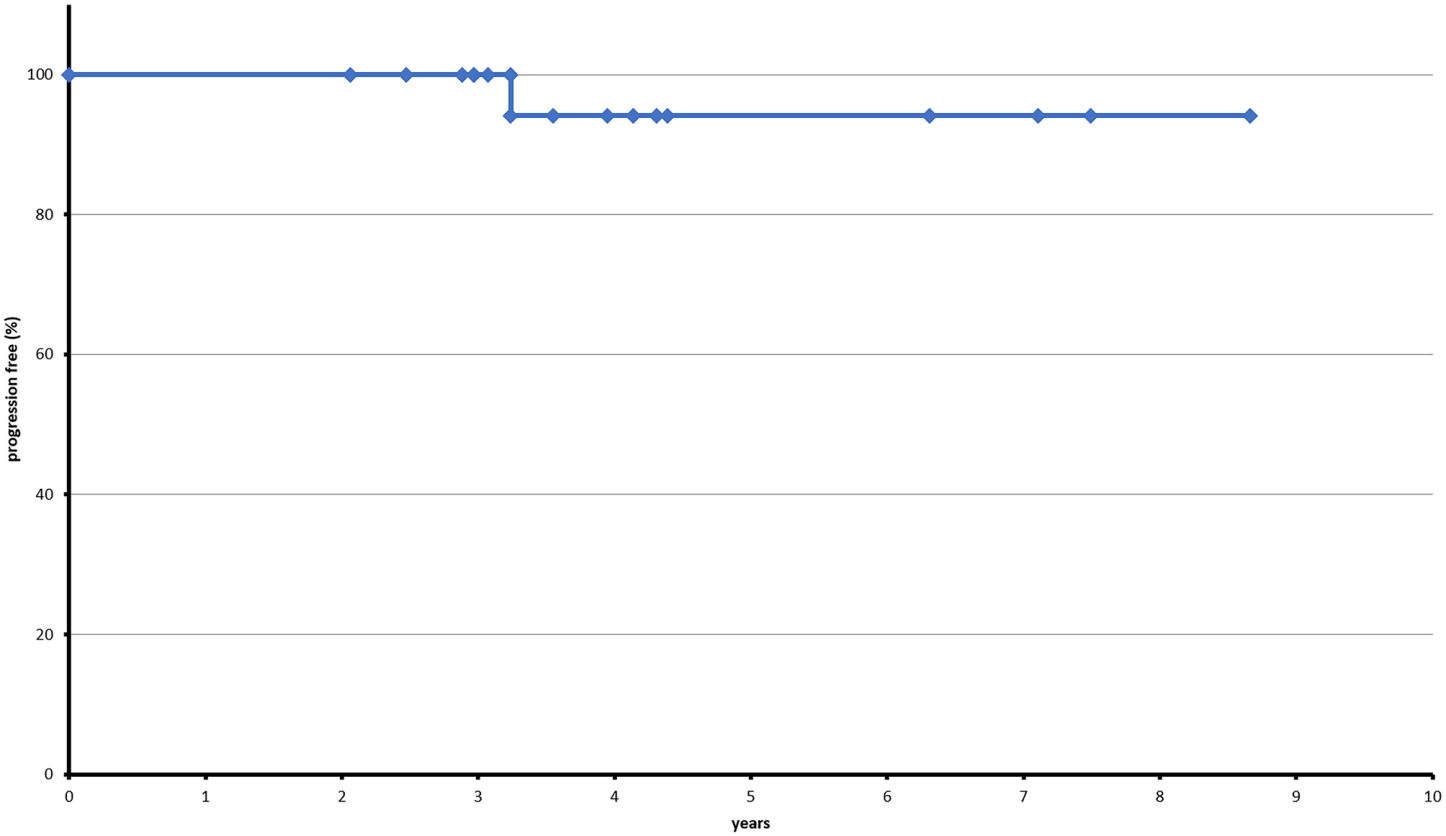
PFS under study protocol. Median follow-up is 4.8 years, currently

Baseline procedures included daily laboratory tests and clinical evaluation during hospital accommodation. In outpatients, weekly controls of liver enzymes, renal function, and blood counts as well as a clinical evaluation were performed. All patients received prophylaxis with Aciclovir 800 mg 2×/day and Cotrimoxazole or Pentacarinate inhalation during the treatment until 60 days after aHSCT. If the patient displayed mucositis grade 3 or higher, prophylaxis with oral Amphotericine B and fluorchinolones was administered. Granulocyte colony-stimulating factor (G-CSF) was administered during periods with < 1000 leukocytes/nl (Fig. 2).

### Adverse events reporting

Toxicity data were retrospectively collected and graded based on the CTCAE 5.0 criteria. For collection, we analyzed all laboratory values before, during and after treatment, viewed all day-to-day clinical documentation and systematically consulted the patients about long-term effects and toxicities every 3–6 months.

## Results

### Patient characteristics

**Fig. 3:**
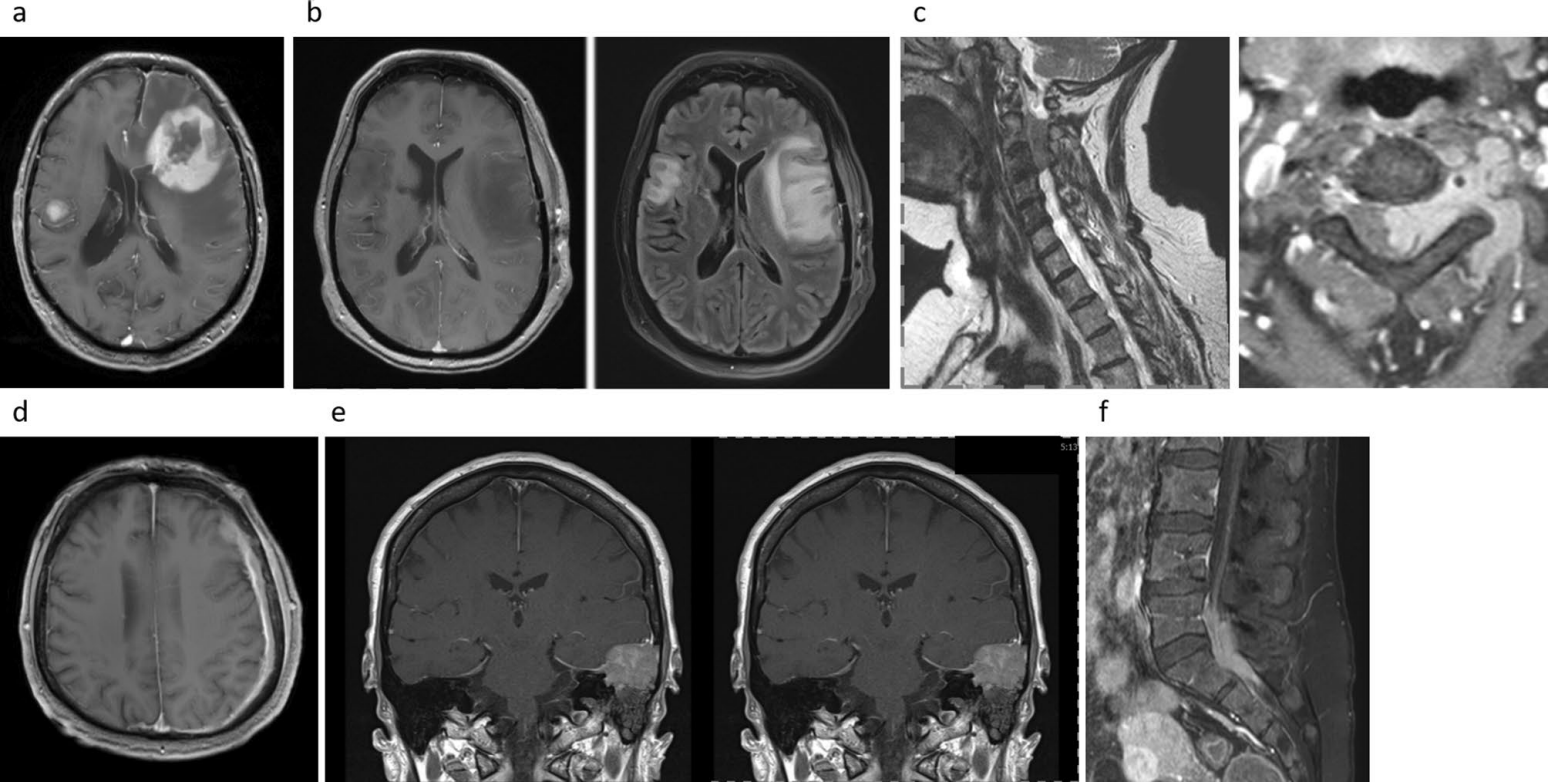
Radiologic aspects of central lymphoma manifestations in our study. **a** Cerebral DLBCL. T1-weighted (T1-w) 2D FLASH sequence post-contrast. **b** Intravascular LBCL of small- and medium-sized intracerebral and meningeal vessels. T1-weighted gradient-recalled echo (GRE) post-contrast and T2 FLAIR TSE BLADE sequence. **c** Spinal dura infiltration of DLBCL. T2-weighted turbo spin-echo (TSE). T1 fat saturated turbo spin-echo (TSE) post-contrast. **d** Cerebral dura infiltration of DLBCL. T1-weighted (T1-w) 2D FLASH sequence post-contrast. **e** Bone-associated lymphoma with cerebral infiltration. T1-weighted (T1-w) Turbo Spin-Echo (TSE) sequence post-contrast. **f** Spinal dura infiltration of DLBCL. Fat-saturated T1-weighted (T1-w) Turbo Spin-Echo (TSE) sequence post-contrast

Overall, 16 patients with recently diagnosed PCL, treated between March 2013 and June 2018, are included in the analysis. Median age was 60.2 years, 50% of them were male and most patients had high–intermediate (31.3%) or high-risk (37.5%) lymphoma according to aggressive NHL IPI stratification. Three patients were graded ECOG 2. One patient was enrolled despite an ECOG 3 status, which was due to cerebral lymphoma. All other patients enrolled (75%) had an ECOG performance status of 1. MYC status was negative where retrospective FISH analysis was possible. CNS involvement presented as cerebral infiltration in six patients, and spinal lymphoma infiltration was revealed in another six patients. In three patients, MRI revealed dura infiltration. One patient had a clinically evident central Horner's syndrome without possible MRI verification. Two patients had been irradiated on the right arm and duodenal bulb, respectively, with 40 Gy shortly before diagnosis of central lymphoma involvement. CSF lymphoma cells were detected in two patients, three patients did not undergo lumbar puncture at initial diagnosis. Patient characteristics are summarized in Table 2. Figure 3 shows central nervous infiltration types as diagnosed via MRI.

**Table 2:** Disease characteristics, therapies, and outcomes by patient

Pat #	Age	Sex	Histology	CNS involvement	CNS infiltration diagnosed via	aIPI	ECOG	Outcome Ping-Pong	Outcome aHSCT	Dose reduction	Additional therapy
1	34.7	M	DLBCL	Cerebral	Clinical course	3	1	PR	CR	No	No
2	57.3	F	DLBCL (from FL)	Cerebral	Biopsy	1	1	CR	CR	No	1 × Depocyte
3	67.2	F	DLBCL (from FL)	Spinal	Biopsy	3	1	CR	CR	Only 3 × MTX	No
4	48.1	F	DLBCL	Dura infiltration	Biopsy	1	1	PR	CR	No	No
5	64.7	M	DLBCL	Spinal	Biopsy	2	2	PR	CR	2 g/m^2^ MTX from c3	3 × intrathecal triple
6	50.8	F	Intravasc. LBCL	Cerebral	Biopsy	3	3	CR	CR	Only 3 × MTX	No
7	56.4	M	DLBCL (HIV-ass.)	Spinal	MRI	3	1	PR	PD	No	Prephase glucocorticoids, radiation (12 Gy brachial plexus) 1 month before
8	68.4	M	DLBCL	Dura infiltration	MRI	2	1	PR	CR	No	No
9	64.2	F	DLBCL (from FL)	Cerebral	Biopsy	1	1	PR	CR	No	No
10	37.1	M	DLBCL	Cerebral	MRI	2	1	CR	CR	Only 1 × MTX	No
11	71.9	F	DLBCL	Spinal	MRI	2	1	PR	CR	No	1 × intrathecal triple
12	65.1	M	DLBCL (from MCL)	Dura infiltration	MRI	3	2	PR	CR	No	Radiation (40,6 Gy duodenal bulb) 3 months before systemic therapy
13	76.7	M	DLBCL	Cerebral	MRI	3	2	PR	CR	2 × Cyclophosphamide 75%	Radiation (40 Gy, right arm) 1 year before systemic therapy
14	74.7	M	DLBCL	Spinal	MRI	1	1	PR	CR	Only 2 × MTX	No
15	69.0	F	DLBCL	Cerebral	Biopsy	1	1	PR	CR	No	3 × Depocyte
16	61.3	F	DLBCL	Spinal	Biopsy	2	1	PR	CR	No	Radiation (PET-guided, 40 Gy mesenterial root/greater trochanter, post-Tx)

All remission classification according to Lugano criteria. Abbreviations: *PR* partial remission, *CR* complete remission, *MTX* methotrexate, *M* male, *F* female, *DLBCL* diffuse large B-cell lymphoma, *FL* follicular lymphoma, *LBCL* large B-cell lymphoma

### Toxicity and dose reduction of Ping-Pong protocol

All 16 patients received the first block of chemotherapy consisting of four cycles of R-CHOP and R-MTX (see Fig. 1). Age-adjusted Vincristine dose reduction was performed according to R-CHOP guidelines. In nine patients (56.3%), no further dose reduction was required. All patients developed grade 3/4 adverse events according to CTCAE. Acute kidney failure also occurred in 31.3% of the patients, all of which were considered MTX-associated. One patient received only 50% of the MTX dose after acute kidney failure during the second R-MTX cycle, and two patients discontinued MTX after acute renal failure or high-grade mucositis. Another patient suffered kidney failure, which remained a grade 3 chronic renal impairment. All other renal failures resolved without sequelae. None of the patients died due to treatment-related toxicity or complications. Grade 3 to 4 hematotoxicity was observed in all patients. On average, duration of the four cycles was 13.5 weeks, compared to a theoretical minimum of 12 weeks, suggesting good tolerability. Prolonged therapy duration was due to infections in all eight cases (50%). During Ping-Pong, three of the patients required platelet transfusion, and 5 out of 16 received red blood cell transfusions. Significant liver enzyme elevations occurred in four cases (25%) and were completely reversible. Severe complications included port infections in three cases (18.8%) and Clostridium difficile-associated diarrhea in two cases. Table 3 presents an overview of grades 3–4 complications. No deaths occurred.

**Table 3:** Grade 3/4 complications during R-CHOP/R-MTX and AraC/TT and HD with aHSCT (CTCAE)

Complication	During Ping-Pong	During AraC/TT + HD
Acute kidney failure (via RIFLE criteria)	5 (31.3%)	0
Grade 3/4 anemia	5 (31.3%)	11 (73.3%)
Grade 3/4 thrombopenia	4 (25.0%)	14 (93.3%)
Febrile neutropenia	4 (25.0%)	7 (46.7%)
Elevated liver enzymes	4 (25.0%)	2 (13.3%)
Port infection	3 (18.8%)	0
Clostridium difficile-associated diarrhea	2 (12.5%)	1 (6.7%)
Mucositis	2 (12.5%)	0
Urinary tract infection	2 (12.5%)	1 (6.7%)
EBV/CMV/HSV reactivation	1 (6.3%)	0
Candida infection	1 (6.3%)	0
Delirium	0	2 (13.3%)
Sepsis	0	1 (6.7%)

*EBV* Epstein–Barr Virus, *CMV* cytomegaly virus, *HSV* herpes simplex virus

All patients qualified for the intensification with two cycles of R-AraC/TT with stem cell harvest after the first cycle and HD-BCNU/TT followed by aHSCT, a regimen often used in primary CNS lymphoma (see Fig. 1). One patient received radiation (12 Gy) of the brachial plexus due to lymphoma-associated sensomotoric impairment during this block of therapy.

### Response after Ping-Pong protocol

We used a combination of CT scan and MRI of the involved region to evaluate response in all 16 patients enrolled after four cycles R-CHOP/R-MTX.

According to IWG 2007 response criteria, all patients achieved a response, resulting in an ORR of 100%. This includes 4 (25%) complete remissions (CR) and 11 partial remissions (75%). We observed no stable or progressive disease under the Ping-Pong protocol.

### Treatment-emergent adverse events of AraC/TT and high-dose chemotherapy

15 of the patients included proceeded to AraC/TT and subsequent HD with aHSCT (see Fig. 1) consisting of 2 cycles AraC/TT and HD-BCNU/TT.

This block was applied during a medium of 10, 6 weeks, compared to a minimum duration of 10 weeks, suggesting good tolerability. At least 4 × 10^6^/kg CD34^+^ cells for each of the two transplants were collected after the first cycle of R-AraC/TT. However, three patients (18.75%) required Plerixafor application to mobilize a sufficient number of stem cells.

14 patients (93.3%) required platelet transfusions, and 11 (73.3%) red blood cell transfusions after HD. In seven, febrile neutropenia occurred, which proved well manageable. Severe complications included medication-associated deliria in two cases (13.3%) and sepsis with ICU transfer in one case (6.7%). No dose reductions were necessary. All patients were able to complete the protocol and qualified for AraC/TT and HD with aHSCT. None of the patients died due to treatment-related toxicity or complications. Grades 3–4 hematotoxicity was observed in all patients. 13 of the patients developed infectious complications grade 3 or higher. An overview of grades 3–4 complications is given in Table 3. Median time to neutrophil regeneration was 8.2 days.

### Response after successive AraC/TT and high-dose chemotherapy protocol

Ping-Pong was conducted to prepare the enrolled patients for the local standard of care protocol including two cycles of R-AraC/TT with stem cell harvest after the first cycle and HD-BCNU/TT followed by aHSCT (see Fig. 1). 15 patients (93.75%) reached CR in cMRI and thoracoabdominal CT scan combined. One patient (6.25%) showed signs of progression during the first cycle of R-AraC/TT after response to Ping-Pong protocol and was not continued, showing a PFS of 4 months. Histologically, he suffered from HIV-related lymphoma which has historically been linked to worse prognosis and therapy outcome (Diamond et al. 2006).

Table 2 outlines all therapy modifications made. All patients who had undergone aHSCT achieved lasting CR. Although median PFS and OS survival have not been reached after 4.8 years. So far, we have not experienced relapses. One patient required PET-guided consolidating radiation (40 Gy) of the trochanter region.

### PFS, DOR, and overall survival

Current median follow-up is 4.8 years (see Fig. 2). So far, no further relapses were observed. The overall survival rate is at 93.25%, currently. Correspondingly, overall survival for transplanted patients is at 100%. Follow-up visits take place every 3 months for the first 3 years, then every 6 months until 5 years after HD.

### Long-term toxicities

During follow-up after therapy completion, patients were systematically interviewed about long-term effects every 3–6 months. Most commonly, patients complained about fatigue (40%). Other problems reported were polyneuropathy and cognitive impairment. Three patients reported persistence of problems caused by irreversible damage caused by lymphoma manifestations. These include paraneoplastic stiff man syndrome, blindness, and back pain, each 6.7%, respectively. One patient suffered chronic renal impairment and heart failure, and hematopoietic recovery was incomplete in another patient. Six patients did not report any sequelae. None of the patients received further therapy or developed a secondary malignancy to this point.

## Discussion

Central nervous involvement is a rare and adverse presentation in lymphoma, offering significantly worse outcomes. Various approaches exist to optimize therapy regimen. In 2015, the LYSA and LOC network published results of a retrospective 60-case study on heterogeneous up-front treatments for SCL. Here, an overall progression-free 3-year-survival of 40–50% was reported after HD with aHSCT, suggesting an important role for HD consolidation therapy in this subset of patients (Damaj et al. 2015). However, Wight et al. did not observe a statistically significant improvement in 2-year PFS after various induction protocols followed by HD compared to no HD (Wight et al. 2019). Current protocols for primary central nervous lymphoma and relapsed synchronous systemic and central nervous lymphoma offer similar outcomes after HD with aHSCT (El-Galaly et al. 2018; Kasenda et al. 2012). Neither of the studies provide data about complications or tolerability.

Two prospective trials studied a similar patient cohort with concomitant systemic and central nervous lymphoma. In 2015, Ferreri et al. reported a 5-year OS rate of 41% with combination therapy including HD-MTX, HD cytarabine, and HD etoposide combined with rituximab and liposomal cytarabine followed by BCNU/TT consolidation and aHSCT. The study included 16 PCL patients, but does not provide data about the specific subgroup (Ferreri et al. 2015a).

The MARIETTA trial reported 32 patients PCL among a greater collective, both relapsed and therapy naive. After three courses of MATRix (AraC, TT and R) as well as three courses of R-ICE (R, ifosfamide, cisplatin, E), intrathecal therapy, and BCNU/TT + aHSCT, a 2-year OS of 42% was reported (Ferreri et al. 2021).

The idea to combine HD-MTX with common therapies for systemic lymphoma is not new; Chihara et al. reported results for EPOCH-R (etoposide, prednisone, vincristine, cyclophosphamide, doxorubicin, and R) with mid-cycle HD-MTX. After a median of five cycles, all of the eight patients with PCL achieved CR with an OS of 100% at a median follow-up at 11 months. Four of the patients received consolidating aHSCT (Chihara et al. 2017). Details on toxicities are not given.

This supports our findings, suggesting high effectivity of mid-cycle MTX in PCL and a significant effect of CNS-intensive induction therapy towards acceptable OS results in this challenging patient population. Puckrin et al. recently presented retrospective data on a large cohort of patients with high IPI lymphoma. Here, prophylactic HD-MTX did not reduce the number of CNS relapses, questioning the role for HD-MTX in long-term PFS (Puckrin et al. 2020). However, MTX is considered a backbone for patients with central lymphoma.

In other retrospective studies, several groups reported significantly worse OS rates of roughly 50% in heterogeneously treated cohorts, mostly without HD + aHSCT without giving details on toxicities (Wight et al. 2019; Maciocia et al. 2016; Nijland et al. 2017).

The worse outcomes reported by other groups can be due to the significantly worse performance status in their cohorts. Often, patients did not qualify for HD, which we believe to be a crucial part of successful long-term therapy. Consolidating HD is associated with a lower risk of CNS relapse in high-risk lymphoma (Puckrin et al. 2020). For the prevention of secondary central nervous lymphoma, lack of cerebral low-dose intensity of CNS-effective treatment is a long-known risk factor, and HD with aHSCT has been recommended (Bromberg et al. 2013; Doolittle et al. 2008; Maziarz et al. 2013).

We provide further evidence that consolidating HD with aHSCT, if tolerated by the patient, should be pursued in PCL patients to optimize outcomes. However, we report better OS data than other retrospective reports. Thus, selection biases must be thoroughly discussed.

Therapy outcomes in lymphoma greatly depend on risk stratification. All patients enrolled presented an Ann-Arbor stage IV and had a high–intermediate to high-risk classification according to IPI, suggesting a high-risk patient cohort. However, our protocol is more aggressive than the uniformly applied R-CHOP, and other approaches such as prophylactic HD-MTX did not reduce the number of CNS relapses (Puckrin et al. 2020). This may overcome the impact of high IPI for the prognosis of our cohort.

In addition, none of the 14 analyzed patients had a double or triple hit lymphoma. While the CNS involvement in our cohort comes with high-risk classification, the lack of molecular high-risk patients may constitute a favorable population within this range. Interestingly, although three of the DLBCL were histopathologically suspected to have developed from follicular lymphoma (FL), no MYC alterations were detected via FISH. However, DLBCL transformation from FL can be attributed to other mutations we did not screen for (Fischer et al. 2018).

Only in 8 out of 16 patients, CNS lymphoma infiltration was histologically confirmed as biopsy often proved not feasible, too risky, or without therapeutic consequence. The good outcome in our cohort may be influenced by overtreatment. This may especially be true for the three patients that were enrolled due to lymphoma infiltration of the dura mater. One patient, although MRI-negative, was clinically suspected to have diffuse cerebral lymphoma infiltration, but the initial Horner's syndrome gradually recovered under therapy, suggesting success of CNS-directed therapy. HD is known to reduce the number of relapses in patients with high aIPI (Puckrin et al. 2020), which may solely explain the lack of long-term relapses in our cohort.

This study was performed at our center only. The possible confounding factors limit the value of the data presented.

Notably, other reports included a significantly higher percentage of patients had a performance status of three or higher, which may partly explain the difference in outcome. While our cohort offers far better OS data, it is a retrospective analysis and thus susceptible to selection biases. All patients that received Ping-Pong at our center are included in this study.

Concerning adverse events during induction therapy, only few studies offer detailed information for this specific patient subset. Ferreri et al. reported better tolerability data for the SCNSL1 trial, with mainly hematologic high-grade and rare infectious complications, whereas only 75% of all patients suffered high-grade complications (Ferreri et al. 2015b).

During the MARIETTA trial, SAEs occurred in slightly over 50% of the patients, with hematologic events being the most common (Ferreri et al. 2021). However, only 53% qualified for planned HD and aHSCT. Four therapy-associated deaths occurred in both trials.

Maciocia et al. reported in individualized consolidation therapy regimens with and without HD and aHSCT after 1–4 cycles R‐IDARAM significantly less-grade 3/4 neutropenia, anemia, and thrombocytopenia (55, 62, and 12%, retrospectively). Hepatotoxicity occurred in 14% of cycles (Maciocia et al. 2016).

However, we observed grade 3/4 toxicities in all patients. Notably, the underestimation of side effects is a major limitation of our retrospective study and should be considered when comparing data, suggesting an even higher toxicity of Ping-Pong with HD consolidation, and may point to a more aggressive regimen at our center towards a relatively healthy population. No therapy-associated deaths occurred during our protocol, which may solely be due to the low number of patients treated.

Complication rates during AraC/TT and stem cell harvest after Ping-Pong are similar to current high-dose concepts of central nervous lymphoma (Illerhaus et al. 2006). Average duration of aplasia during HD was comparable at 8.5 days, suggesting adequate dosing and usual aHSCT complication patterns. In all patients, stem cell mobilization was successful. However, a significant number required Plerixafor application to mobilize a sufficient number of stem cells. The relatively high dosage of chemotherapy compared to other concepts before stem cell harvest may play a role here. Other groups reported no mobilization issues after HD-MTX and R-AraC/TT in PCNSL patients (Kasenda et al. 2012; Cheng et al. 2003).

This study provides a promising approach to the rare and difficult-to-treat entity of systemic lymphoma with primary CNS involvement. At our center, R-CHOP and HD-MTX with HD and aHSCT were feasible. Our data suggest effectiveness for transplant-eligible patients with concomitant peripheral and central nervous B-cell lymphoma. Provided the low incidence, not many patients were eligible for this study. Protocol application will proceed, given these promising preliminary results.

## Data Availability

The data that support the findings of this study are available from the corresponding author upon request. The authors declare that all data supporting the findings of this study are available within the paper.
